# Low Rates of Repeat HIV Testing Despite Increased Availability of Antiretroviral Therapy in Rural Tanzania: Findings from 2003–2010

**DOI:** 10.1371/journal.pone.0062212

**Published:** 2013-04-23

**Authors:** Caoimhe Cawley, Alison Wringe, Raphael Isingo, Baltazar Mtenga, Benjamin Clark, Milly Marston, Jim Todd, Mark Urassa, Basia Zaba

**Affiliations:** 1 Department of Population Health, London School of Hygiene and Tropical Medicine, London, United Kingdom; 2 TAZAMA Project, National Institute for Medical Research, Mwanza, Tanzania; Alberta Provincial Laboratory for Public Health/University of Alberta, Canada

## Abstract

**Background:**

HIV counselling and testing (HCT) services can play an important role in HIV prevention by encouraging safe sexual behaviours and linking HIV-infected clients to antiretroviral therapy (ART). However, regular repeat testing by high-risk HIV-negative individuals is important for timely initiation of ART as part of the ‘treatment as prevention’ approach.

**Aim:**

To investigate HCT use during a round of HIV serological surveillance in northwest Tanzania in 2010, and to explore rates of repeat testing between 2003 and 2010.

**Methods:**

HCT services were provided during the fourth, fifth and sixth rounds of serological surveillance in 2003–2004 (Sero-4), 2006–2007 (Sero-5) and 2010 (Sero-6). HCT services have also been available at a government-run health centre and at other clinics in the study area since 2005. Questionnaires administered during sero-surveys collected information on socio-demographic characteristics, sexual behaviour and reported previous use of HCT services.

**Results:**

The proportion of participants using HCT increased from 9.4% at Sero-4 to 16.6% at Sero-5 and 25.5% at Sero-6. Among participants attending all three sero-survey rounds (n = 2,010), the proportions using HCT twice or more were low, with 11.1% using the HCT service offered at sero-surveys twice or more, and 25.3% having tested twice or more if reported use of HCT outside of sero-surveys was taken into account. In multivariable analyses, individuals testing HIV-positive were less likely to repeat test than individuals testing HIV-negative (aOR 0.17, 95% CI 0.006–0.52).

**Discussion/Conclusions:**

Although HCT service use increased over time, it was disappointing that the proportions ever testing and ever repeat-testing were not even larger, considering the increasing availability of HCT and ART in the study area. There was some evidence that HIV-negative people with higher risk sexual behaviours were most likely to repeat test, which was encouraging in terms of the potential to pick-up those at greatest risk of HIV-infection.

## Introduction

HIV counselling and testing (HCT) services have recently expanded rapidly in sub-Saharan Africa in order to facilitate access to HIV treatment and care [1]. It has also been widely assumed that HCT services play an important role in HIV prevention, by motivating both HIV-negative and HIV-positive individuals to reduce sexual risk taking and adopt preventive behaviours [2], [3]. The contribution of HCT to HIV prevention may potentially acquire a further role in light of the treatment as prevention (TasP) approach, which suggests that widespread use of antiretroviral therapy (ART) could have a substantial impact in reducing rates of HIV transmission as a result of reduced viral loads in treated patients [4]–[6]. One modelling study suggested that regular testing for all adults aged 15 and over once per year, followed by immediate access to treatment for those testing positive, could result in the near elimination of HIV in generalised epidemic settings within 50 years [4]. However, this assertion has been challenged by other authors [7].

Despite increases in the numbers of individuals accessing HIV testing and treatment services, the World Health Organisation reported in 2011 that knowledge of HIV status remains broadly inadequate. In six countries with results from population-based surveys conducted in 2007–2009, the proportion of respondents unaware of their HIV-positive status ranged from about 30% in Kenya to almost 70% in the Congo [1]. Only a few studies have investigated rates of repeat testing in sub-Saharan Africa [8]–[11], with just two of these being carried out since ART has become more widely and freely available [8], [9]. In terms of eventual TasP implementation, it would be particularly important that individuals initially testing HIV-negative but who are at high risk of sero-conversion (for example as a result of high risk sexual behaviour) come back to test on a regular basis.

This paper reports on the uptake of HCT services during the latest round of HIV serological surveillance carried out in 2010 as part of an on-going community cohort study in Kisesa in northwest Tanzania. It also reports on repeat use of HCT services over consecutive rounds of serological surveillance carried out between 2003 and 2010; a period of time which saw a number of changes in terms of the availability of HIV services, including the commencement of a national ART programme at the start of 2005, and the opening of a permanent HCT clinic in the study area later that year. We explore how these changes affected rates of first time and repeat HCT over time.

## Methods

### Ethical Statement

Ethical approval for each survey round of the Kisesa cohort study has been granted by the Tanzanian Medical Research Coordinating Committee and the Ethics Committee of the London School of Hygiene and Tropical Medicine. During the 2003–2004 survey, verbal consent was obtained directly from all study participants including those aged 15–17, due to low literacy rates among the study population. Consent was witnessed and documented for each study participant on their survey questionnaire, by a member of the sero-survey team. During serological surveys in 2006–2007 and 2010, consent was again obtained directly from all study participants including those aged 15–17, however the option of written consent was introduced, for those who were able to provide this. Although data from the subsequent serological survey in 2012–2013 are not used in this paper, it was recommended by the review committee that additional written consent be obtained from parents or guardians for participants aged 15–17 for this round, and this was implemented accordingly.

### Study Setting

The study setting in Mwanza region has been described in detail by Mwaluko *et al* [12]. Briefly, the study area lies approximately 20 km to the east of Mwanza city and consists of six villages which make up the administrative ward of Kisesa, with a combined population of approximately 32,000 people. Some sub-villages lie along the main tarmac road which cuts through the study area and leads to the border with Kenya, while other sub-villages are located further away from the road in rural areas. The largest village in the study area, Kisesa trading centre, lies directly along the main road.

Since 1994, 27 rounds of demographic surveillance and six HIV serological surveys have been completed. Demographic surveillance took place approximately once every six months, collecting information on residence and survival status of all household members, pregnancy among women of reproductive age, and births and migration. Eligibility and invitations for the serological surveys (sero-surveys) were prepared based on having been resident at the last round of demographic surveillance and being aged 15 or older at the time of the sero-survey. Venous blood was collected from all consenting participants during the first sero-survey in 1994/1995, while finger prick blood was collected during all subsequent sero-surveys. Samples were tested for HIV anonymously at the National Institute for Medical Research (NIMR) in Mwanza.

A separate and confidential HCT service was provided in temporary village based facilities during the fourth, fifth and sixth rounds of serological surveillance in 2003–2004 (Sero-4), 2006–2007 (Sero-5) and 2010 (Sero-6), for those wishing to know their HIV status (see below for further details). Height and weight measurements were taken during sero-surveys, accompanied by a detailed questionnaire. The questionnaire was administered by same sex interviewers and included items on socio-demographic characteristics, sexual behaviour, health status and previous HCT service use. All study participants were additionally offered free medical treatment for any health problems present at the time of the sero-survey.

### HIV Counselling and Testing and other Health Services

The study population is served by a government-run health centre located in Kisesa trading centre, by three small government-run dispensaries located in the rural villages, and by a number of private clinics located mainly in the trading centre. HCT services are distinguished as those provided at stand-alone voluntary counselling and testing (VCT) clinics, or via other clinics within a hospital or health centre (usually at antenatal, sexually transmitted infection or tuberculosis clinics), referred to as provider initiated testing and counselling (PITC). A stand-alone VCT clinic opened at Kisesa health centre in 2005, while PITC has been offered to all pregnant women attending the health centre antenatal clinic (ANC) since the end of 2008. PITC has also been available at the out-patients department since 2010, where testing may be offered to patients attending the sexually transmitted infection and tuberculosis clinics. Since mid-2009, antenatal PITC is usually offered at the small rural dispensaries, dependant on the availability of test-kit supplies.

When HCT services were first provided in Kisesa in 2005, patients testing HIV-positive were referred to Mwanza city hospitals, where free care and treatment services have been available under the national ART programme since the beginning of 2005. At the end of 2008, ART services became available locally at a Care and Treatment Centre (CTC) at Kisesa health centre. At this point, individuals testing HIV-positive were referred to Kisesa CTC for care, while patients already attending Mwanza city hospitals were given the option of transferring to Kisesa CTC if they wished.

Since the fourth sero-survey round in 2003–2004, a separate and temporary VCT clinic has been available to all sero-survey participants wishing to know their HIV status. After their questionnaire interview, individuals expressing desire for VCT were directed to a separate purpose-constructed hut for pre-test counselling with a trained counsellor. At Sero-4 venous blood was collected and transported to the NIMR laboratory for HIV testing, and clients were asked to return for their test results and post-test counselling one week later [13]. During Seros 5 and 6, venous blood was again collected but rapid HIV screening tests were used; results and post-test counselling were usually delivered within 45 minutes of the test being performed [14]. Individuals testing HIV-positive at Sero-4 were informed that treatment would become available in the near future through the national ART programme. With their prior agreement, these individuals were subsequently traced by the VCT counsellors and referred to the zonal referral hospital in Mwanza. Individuals testing HIV-positive at Sero-5 were referred directly to Mwanza city hospitals, while those testing positive at Sero-6 were referred to the local Kisesa CTC.

### Data and Analysis

All survey data were double entered. Linking of demographic data, sero-survey interview data, HIV research tests and sero-survey VCT attendance was carried out anonymously using study identification numbers. Sexual behaviour variables collected during sero-survey interviews included age at first sex, number of sexual partners and frequency of condom use with different partner types. Marital change and spouse identification variables were obtained from the demographic data, after which spouses’ HIV-status was anonymously linked in from the serological data. The percentage change in body mass index (BMI) at each sero-survey was calculated using height and weight data collected at the previous sero round, for those who had attended the previous sero-survey.

At Seros 4, 5 and 6, the survey questionnaire also captured information on participants’ self-reported prior use of HCT services, including VCT use at various other clinics within and outside the study area (e.g. Kisesa health centre VCT, ‘Angaza’ VCT clinics run by the African Medical Research Foundation, other temporary mobile VCT clinics provided occasionally in the study area by non-governmental organisations or regional hospitals), and via PITC at antenatal clinics and/or hospital outpatient departments. At Seros 5 and 6, it was possible to additionally identify those with known prior use of VCT at an earlier sero-survey round, for those who had attended an earlier round.

All statistical analyses were done using Stata 12 (StataCorp LP, Texas, USA). In order to ensure that all individuals had an equal period of time during which they might have repeat tested, a repeat tester was defined as somebody who attended both Sero-5 and Sero-6 and who used the VCT service available at each of these rounds. Univariable analyses were used to describe associations between all variables of interest and i) uptake of VCT at the Sero-6 round, ii) repeat use of VCT at the Sero-5 and Sero-6 rounds. Logistic regression models were fitted to identify characteristics independently associated with VCT uptake using a forward-fitting approach and including all variables significant in univariable analyses at the p≤0.10 level. Likelihood ratio tests were used to assess the inclusion of variables in multivariable models.

## Results

### Factors Associated with VCT Use at Sero-6

Altogether 8,008 individuals attended the Sero-6 round, of whom 60.9% were women. In total 31.7% of men and 30.5% of women gave consent to use VCT, while 25.9% of men and 25.2% of women subsequently went for VCT. This represents approximately a doubling in the proportion of men using VCT since Sero-4, and a threefold increase amongst women (Table 1).

**Table 1:** Univariable analysis of factors most strongly associated with VCT uptake at sero-surveys 4, 5 and 6^¤.^

		Sero-4			Sero-5			Sero-6		
		No.	% using VCT	OR (95% CI)	No.	% using VCT	OR (95% CI)	No.	% using VCT	OR (95% CI)
**MEN - Total**		**3,978**	**12.1**		**3,633**	**18.3**		**3,131**	**25.9**	
Area of residence	Rural	2,109	10.3	1	2,052	16.7	1	1,772	15.8	1
	Roadside	991	11.9	1.18 (0.93–1.49)	940	20.7	**1.31 (1.08–1.59)** *	763	36.3	**3.04 (2.50–3.69)** *
	Trading Centre	878	16.7	**1.75 (1.40–2.20)** *	641	19.8	**1.24 (0.99–1.55)** ¶	596	42.8	**3.98 (3.24–4.90)** *
Education	None	623	4.0	**0.26 (0.17–0.39)** *	670	13.0	**0.62 (0.48–0.80)** *	453	18.1	**0.61 (0.47–0.79)** *
	Primary 1–4 years	794	9.2	**0.62 (0.48–0.81)** *	445	14.8	**0.73 (0.55–0.97)** *	324	25.3	0.93 (0.71–1.23)
	Primary 5–7 years	2,216	14.0	1	1,966	19.3	1	1,563	26.7	1
	Secondary or higher	344	21.1	**1.63 (1.23–2.17)** *	546	24.0	**1.32 (1.05–1.65)** *	777	29.1	1.13 (0.93–1.36)
HIV status	Negative	3,709	11.8	1	3,445	17.8	1	2,947	25.9	1
	Positive	213	17.8	**1.63 (1.13–2.35)** *	173	28.3	**1.83 (1.30–2.58)** *	161	28.0	1.11 (0.78–1.58)
Previous HCT use^α^	No	3,893	11.5	1	3,077	15.2	1	2,215	18.2	1
	Yes	85	38.8	**4.87 (3.11–7.62)** *	552	35.7	**3.1 (2.54–3.79)** *	916	44.7	**3.63 (3.06–4.30)** *
Spouse HIV status & VCT use^Δ^	No spouse identified	2,792	11.8	1	2,580	16.4	1	2,444	24.9	1
	HIV neg, no VCT	1,064	9.9	**0.81 (0.65–1.03)** ¶	829	17.6	1.09 (0.89–1.34)	484	19.4	**0.73 (0.57–0.93)** *
	HIV pos, no VCT	37	13.5	1.16 (0.45–3.00)	41	24.4	1.64 (0.80–3.38)	29	31.0	1.36 (0.62–3.00)
	HIV neg, used VCT	70	48.6	**7.03 (4.34–11.38)** *	162	49.4	**4.97 (3.59–6.89)** *	163	58.3	**4.22 (3.05–5.84)** *
	HIV pos, used VCT	6	83.3	**37.2** **(4.33–319.36)** *	13	30.8	2.27 (0.69–7.39)	10	60.0	**4.53** **(1.27–16.10)** *
**WOMEN - Total**		**4,982**	**7.4**		**5,063**	**15.3**		**4,877**	**25.2**	
Area of residence	Rural	2,404	5.0	1	2,631	12.4	1	2,493	14.0	1
	Roadside	1,312	9.1	**1.91 (1.47–2.49)** *	1,303	19.3	**1.69 (1.41–2.02)** *	1,284	34.7	**3.27 (2.78–3.84)** *
	Trading Centre	1,266	10.2	**2.17 (1.68–2.82)** *	1,128	17.6	**1.5 (1.24–1.82)** *	1,100	39.5	**4.02 (3.40–4.74)** *
Education	None	1,661	2.4	**0.21 (0.15–0.30)** *	1,972	10.6	**0.53 (0.44–0.63)** *	1,831	17.7	**0.5 (0.43–0.58)** *
	Primary 1–4 years	786	7.1	**0.68 (0.50–0.92)** *	425	15.8	0.83 (0.62–1.10)	314	28.3	0.92 (0.71–1.20)
	Primary 5–7 years	2,338	10.1	1	2,288	18.4	1	2,212	30.1	1
	Secondary or higher	196	18.4	**2 (1.36–2.94)** *	370	20.5	1.14 (0.87–1.50)	510	28.6	0.93 (0.75–1.15)
HIV status	Negative	4,653	7.1	1	4,690	15.0	1	4,502	25.5	1
	Positive	277	11.6	**1.7 (1.15–2.49)** *	341	20.8	**1.49 (1.13–1.96)** *	359	20.9	**0.77 (0.59–1.00)** ¶
Previous HCT use^α^	No	4,919	7.2	1	4,467	13.4	1	2,921	17.3	1
	Yes	63	22.2	**3.69 (2.02–6.74)** *	592	30.4	**2.83 (2.33–3.44)** *	1,956	36.9	**2.8 (2.45–3.20)** *
Spouse HIV status & VCT use^Δ^	No spouse identified	3,769	7.6	1	3,971	15.0	1	4,180	25.3	1
	HIV neg, no VCT	994	3.9	**0.5 (0.35–0.70)** *	782	10.9	**0.69 (0.54–0.88)** *	450	14.0	**0.48 (0.37–0.63)** *
	HIV pos, no VCT	48	6.3	0.81 (0.25–2.62)	56	14.3	0.94 (0.44–2.00)	37	16.2	0.57 (0.24–1.38)
	HIV neg, used VCT	140	25.0	**4.05 (2.71–6.05)** *	227	34.8	**3.02 (2.26–4.02)** *	196	49.5	**2.9 (2.17–3.87)**
	HIV pos, used VCT	15	26.7	**4.42 (1.40–13.96)** *	22	36.4	**3.23 (1.35–7.73)** *	9	44.4	2.37 (0.63–8.83)

¤ Figures for VCT uptake at Seros 4 and 5 reflect an update on previous figures presented by Wringe *et al* [13] and Isingo *et al* [14]. The figures are provided here for context.

* p≤0.05,

¶ p≤0.1.

α Reported or documented (at an earlier sero-survey round) previous HCT use.

Δ Spouse’s HIV status & VCT use at current sero-survey round.

Table 1 shows trends in the factors most strongly associated with uptake of VCT at Seros 4, 5 and 6 in univariable analyses. (Previous analyses of factors associated with VCT uptake at Seros 4 and 5 have been published by Wringe *et al* [13] and by Isingo *et al* [14]. The analyses were updated and provided in Table 1 for context). These included having previously used an HCT service, having a spouse who used VCT during that sero-survey round, area of residence (those living in roadside villages or in the trading centre being more likely to use VCT compared to those living in rural villages) and level of education (those with most education being most likely to use VCT). In addition, those who were HIV-positive were more likely to test compared to HIV-negatives, except at Sero-6, when HIV-positive women were *less* likely to test than HIV-negative women (odds ratio [OR] 0.77, 95% confidence intervals [CI] 0.59–1.00).

A full analysis of the associations between socio-demographic, clinical and behavioural characteristics and VCT use at Sero-6 can be seen in Tables 2 and 3. Similar factors to those mentioned above persisted as independent predictors of VCT use at the Sero-6 round. Among men, these included previous HCT use (adjusted odds ratio [aOR]: 2.30, 95% CI 1.88–2.81), having a spouse who used VCT at Sero-6 (aOR HIV-negative spouse using VCT: 2.90, 95% CI 1.93–4.36; aOR HIV-positive spouse using VCT: 4.31, 95% CI 1.08–17.26), and area of residence (aOR those living in trading centre compared to rural villages: 3.24, 95% CI 2.55–4.12; aOR those living in roadside compared to rural villages: 2.57, 95% CI 2.07–3.19). Similar factors were independently associated with VCT use among women (Tables 2 and 3) with the addition of level of education; women with no education were significantly less likely to use VCT compared to those with 5–7 years of primary education (aOR 0.67, 95% CI 0.55–0.80).

**Table 2:** Crude and adjusted odds ratios for socio-demographic factors associated with VCT uptake at Sero-6.

		Males				Females			
		No.	% using VCT	OR (95% CI)	aOR (95% CI) 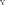	No.	% using VCT	OR (95% CI)	aOR (95% CI) 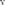
**Total**		**3,131**	**25.9**			**4,877**	**25.2**		
Age	15–24	1,503	19.8	1	1	1,722	23.5	1	1
	25–34	464	37.1	**2.39** **(1.91–3.00)** *	1.09 (0.75–1.59)	1,125	32.4	**1.56** **(1.32–1.84)** *	1.03 (0.83**–**1.29)
	35**–**44	412	35.9	**2.28** **(1.79–2.89)** *	1.17 (0.76**–**1.80)	811	31.2	**1.47** **(1.22––1.78)** *	1.15 (0.90**–**1.47)
	> = 45	751	26.0	**1.42** **(1.16–1.75)** *	0.83 (0.54**–**1.27)	1,219	16.8	**0.66** **(0.55–0.79)** *	0.83 (0.62**–**1.11)
Area of residence	Rural	1,772	15.8	1	1	2,493	14.0	1	1
	Roadside	763	36.3	**3.04** **(2.50–3.69)** *	**2.57** **(2.07–3.19)** *	1,284	34.7	**3.27** **(2.78–3.84)** *	**2.95 (2.45–3.54)** *
	Trading Centre	596	42.8	**3.98** **(3.24–4.90)** *	**3.24** **(2.55–4.12)** *	1,100	39.5	**4.02 (3.40–4.74)** *	**3.39 (2.78–4.15)** *
Ethnicity	Sukuma	2,961	24.7	1	1	4,564	24.4	1	
	Non Sukuma	168	47.6	**2.77** **(2.02–3.79)** *	**1.67** **(1.16–2.40)** *	306	37.3	**1.84** **(1.45–2.35)** *	
Education	None	453	18.1	**0.61** **(0.47–0.79)** *		1,831	17.7	**0.5** **(0.43–0.58)** *	**0.67 (0.55–0.80)** *
	Primary 1**–**4 years	324	25.3	0.93 (0.71**–**1.23)		314	28.3	0.92 (0.71**–**1.20)	0.88 (0.66**–**1.19)
	Primary 5**–**7 years	1,563	26.7	1		2,212	30.1	1	1
	Secondary & higher	777	29.1	1.13 (0.93**–**1.36)		510	28.6	0.93 (0.75**–**1.15)	1.00 (0.74**–**1.34)
Religion	Catholic	1,107	28.2	1		2,062	26.0	1	
	Other Christian	1,416	27.0	0.94 (0.79**–**1.13)		2,433	25.0	0.95 (0.83**–**1.08)	
	Traditional	530	15.8	**0.48** **(0.37–0.63)** *		253	12.3	**0.4** **(0.27–0.58)** *	
	Muslim	75	44.0	**2** **(1.25–3.22)** *		117	42.7	**2.12** **(1.45–3.10)** *	
Marital status	Never Married	1,466	20.1	1	1	1,008	18.3	1	1
	Married monogamous	1,325	31.5	**1.83 (1.54–2.17)** *	0.69 (0.44**–**1.08)	2,448	29.4	**1.86** **(1.55–2.23)** *	0.96 (0.70**–**1.32)
	Marries polygamous	128	37.5	**2.38 (1.63–3.48)** *	0.97 (0.51**–**1.85)	402	29.6	**1.88** **(1.44–2.46)** *	1.07 (0.72**–**1.58)
	Widowed	46	13.0	0.6 (0.25**–**1.42)	**0.43 (0.16–1.18)** ¶	512	13.1	**0.67** **(0.50–0.91)** *	**0.66 (0.42–1.04)** ¶
	Separated/Divorced	97	33.0	**1.95 (1.26–3.04)** *	0.99 (0.53**–**1.88)	464	28.0	**1.74** **(1.35–2.26)** *	1.2 (0.81**–**1.78)
Marital status change	No	2,479	23.5	1	1	3,142	22.7	1	
	Yes	266	31.2	**1.48 (1.12–1.95)** *	1.1 (0.77**–**1.57)	741	26.7	**1.24** **(1.03–1.49)** *	
	Don’t know	386	38.1	**2 (1.60–2.51)** *	**1.7 (1.21–2.39)** *	994	31.7	**1.58** **(1.35–1.85)** *	

* p≤0.05,

¶ p≤0.1.

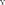
Adjusted for all socio-demographic, clinical & behavioural factors associated with VCT use in univariable analyses (p≤0.10) and remaining significant in multivariable analyses.

**Table 3:** Crude and adjusted odds ratios for clinical and behavioural factors associated with VCT uptake at Sero-6.

		Males				Females			
		No.	% using VCT	OR (95% CI)	aOR (95% CI) 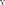	No.	% using VCT	OR (95% CI)	aOR (95% CI) 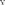
HIV status	Negative	2,947	25.9	1	1	4,502	25.5	1	1
	Positive	161	28.0	1.11 (0.78**–**1.58)	0.72 (0.47**–**1.09)	359	20.9	**0.77** **(0.59–1.00)** ¶	**0.46 (0.34–0.61)** *
BMI loss	None	931	26.9	1	1	1,506	21.8	1	1
	<5%	240	28.7	1.1 (0.80**–**1.51)	1.01 (0.70**–**1.45)	347	21.3	0.97 (0.73**–**1.29)	1.1 (0.81**–**1.51)
	> = 5%	144	18.1	**0.6 (0.38–0.94)** *	**0.55 (0.34–0.91)** *	350	20.9	0.94 (0.71**–**1.25)	1.07 (0.78**–**1.47)
	Unknown	1,816	25.7	0.94 (0.79**–**1.13)	1.09 (0.88**–**1.35)	2,674	28.1	**1.4 (1.20–1.62)** *	**1.64 (1.37–1.96)** *
Previous HCT use^α^	No	2,215	18.2	1	1	2,921	17.3	1	1
	Yes	916	44.7	**3.63 (3.06–4.30)** *	**2.30 (1.88–2.81)** *	1,956	36.9	**2.8 (2.45–3.20)** *	**1.57 (1.33–1.85)** *
Spouse HIV status & VCTuse^Δ^	No spouse identified	2,444	24.9	1	1	4,180	25.3	1	1
	HIV neg, no VCT	484	19.4	**0.73 (0.57–0.93)** *	**0.71 (0.51–0.98)** *	450	14.0	**0.48 (0.37–0.63)** *	**0.55 (0.40–0.74)** *
	HIV pos, no VCT	29	31.0	1.36 (0.62**–**3.00)	1.09 (0.44**–**2.73)	37	16.2	0.57 (0.24**–**1.38)	0.69 (0.28**–**1.74)
	HIV neg, used VCT	163	58.3	**4.22 (3.05–5.84)** *	**2.90 (1.93–4.36)** *	196	49.5	**2.9 (2.17–3.87)** *	**2.17 (1.57–3.00)** *
	HIV pos, used VCT	10	60.0	**4.53 (1.27–16.10)** *	**4.31** **(1.08–17.26)** *	9	44.4	2.37 (0.63**–**8.83)	2.7 (0.66**–**11.07)
Age at first sex	≥15	1,782	33.5	1	1	3,431	30.0	1	1
	<15	256	21.5	**0.54 (0.40–0.74)** *	**0.66 (0.47–0.94)** *	467	20.6	**0.6 (0.48–0.77)** *	**0.73 (0.56–0.95)** *
	Never had sex	825	11.2	**0.25 (0.20–0.32)** *	**0.25 (0.17–0.36)** *	612	8.7	**0.22** **(0.17–0.30)** *	0.51 (0.10**–**2.68)
	Don’t know	237	27.4	**0.75 (0.55–1.01)** ¶	0.78 (0.55**–**1.10)	337	13.9	**0.38** **(0.28–0.52)** *	**0.59 (0.41–0.83)** *
No. of sex partners in last year	Zero	1,650	29.5	1		4,130	27.2	1	1
	One	398	36.2	**1.36 (1.08–1.71)** *		76	46.1	**2.28 (1.44–3.60)** *	**1.99 (1.19–3.31)** *
	Two or more	247	36.4	**1.37 (1.04–1.82)** *		24	37.5	1.6 (0.70**–**3.67)	1.21 (0.47**–**3.13)
Frequency of condom use^Ω^	Never use	1,348	30.9	1	1	2,719	28.5	1	1
	Inconsistent	304	43.8	**1.74 (1.35–2.25)** *	1.21 (0.91**–**1.63)	532	41.9	**1.81** **(1.50–2.20)** *	1.17 (0.93**–**1.47)
	Consistent	86	36.0	1.26 (0.80**–**1.99)	0.84 (0.49**–**1.45)	48	31.3	1.14 (0.62**–**2.11)	0.67 (0.34**–**1.35)
	Never had sex	825	11.2	**0.28 (0.22–0.36)** *	**–**	612	8.7	**0.24** **(0.18–0.32)** *	**–**
	Don’t know	568	24.6	**0.73 (0.59–0.92)** *	**0.72 (0.53–0.98)** *	966	16.8	**0.51** **(0.42–0.61)** *	**0.71 (0.52–0.98)** *
Has a relativewho is HIV**–**positive	No	2,133	24.3	1		3,478	22.6	1	1
	Yes	479	38.8	**1.97 (1.60–2.43)** *		1,148	34.2	**1.79** **(1.54–2.07)** *	**1.19 (0.98–1.42)** ¶
	Don’t know	274	29.2	**1.28 (0.97–1.69)** ¶		199	24.1	1.09 (0.78**–**1.52)	0.85 (0.57**–**1.26)
Knows somebody taking ART	No	1,523	23.4	1		3,029	22.0	1	1
	Yes	935	35.4	**1.79 (1.50–2.14)** *		1,515	34.5	**1.87** **(1.63–2.14)** *	**1.15 (0.96–1.37)** *
	Don’t know	423	22.5	0.95 (0.73**–**1.22)		269	13.8	**0.57** **(0.40–0.81)** *	**0.66 (0.44–0.99)** *

* p≤0.05,

¶ p≤0.1.

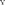
Adjusted for all socio-demographic, clinical & behavioural factors associated with VCT use in univariable analyses (p≤0.10) and remaining significant in multivariable analyses.

α Reported or documented (at an earlier sero-survey round) previous HCT use.

Δ Spouse’s HIV status & VCT use at current sero-survey round.

Ω Frequency of condom use in last 12 months considering three most recent partners.

In contrast to earlier rounds, women who were HIV-positive at Sero-6 were significantly *less* likely to use VCT compared to HIV-negative women (Table 3: aOR 0.46, 95% CI 0.34–0.61). A similar trend was seen among men, although the result was not statistically significant (aOR 0.72 95% CI 0.47–1.09). A closer investigation revealed an interaction between HIV status and previous HCT use. HIV-positive individuals were more likely to use VCT if they had *not* previously used any HCT service (OR men: 1.62, 95% CI 0.99–2.64; OR women: 1.16, 95% CI 0.77–1.75) but not if they *had* previously used HCT (OR men: 0.51, 95% CI 0.30–0.85; OR women: 0.45, 95% CI 0.32–0.63).

### Lifetime use of HCT Services among those Attending Sero-4, 5 and/or 6

**Figure 1:**
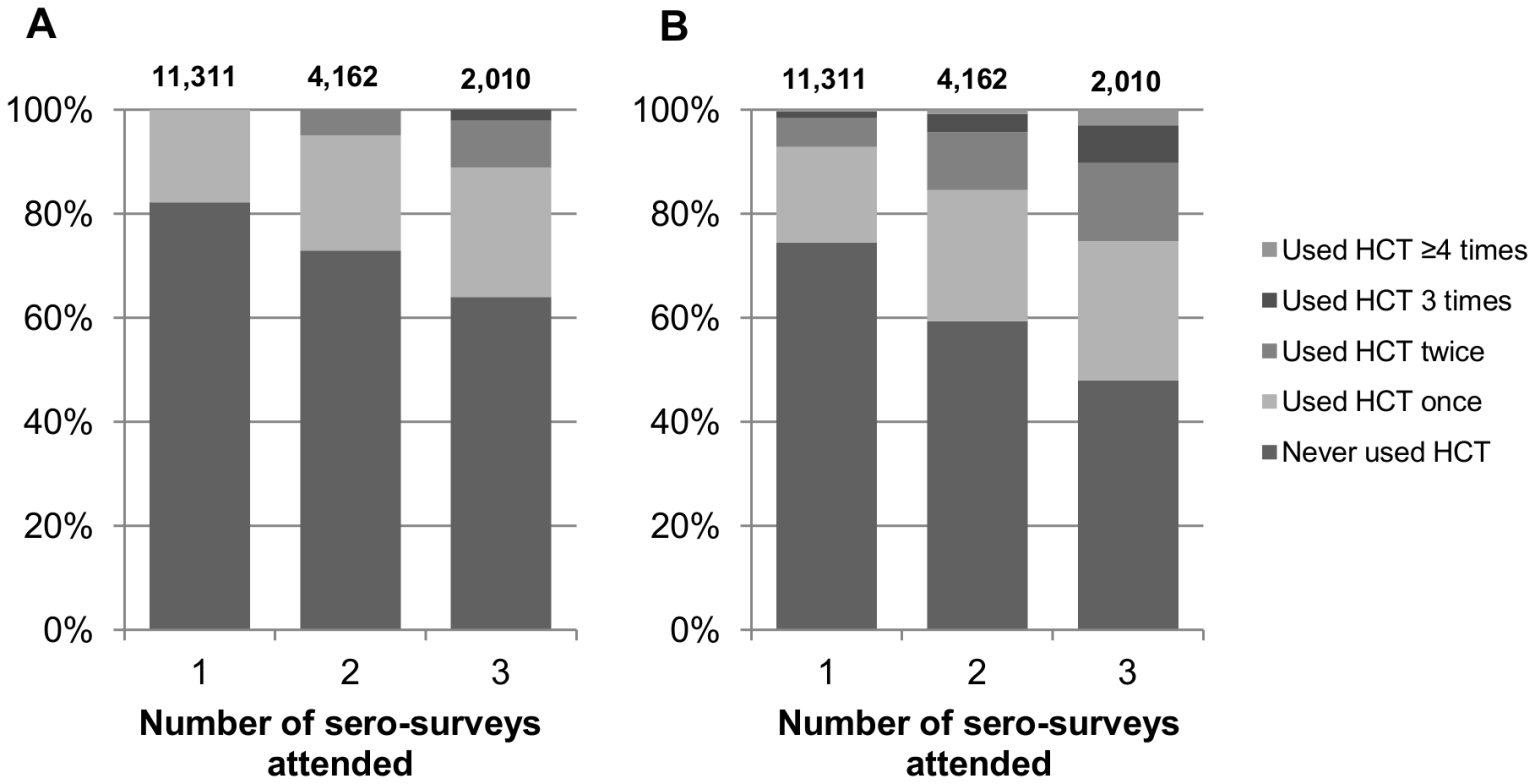
Number of individuals attending one or more of Sero-4, 5 and/or 6 and the number of times HCT was used. Panel A) number of times HCT used during sero-surveys; panel B) number of times HCT ever used (i.e. during sero-surveys *and/or* reported use of HCT outside sero-surveys).

In total over the period 2003–2010, 17,483 individuals attended at least one of Seros 4, 5 and/or 6. Figure 1 shows the number of individuals attending one or more sero-surveys and the number of times a) VCT was used at a sero-survey; b) an HCT service was ever used (i.e. at sero-survey *and/or* reported use of HCT outside sero-surveys). Considering VCT use at sero-surveys only (Figure 1a), of those attending all 3 seros (n = 2,010) 64.0% never used VCT, 24.9% used VCT once and 11.1% used VCT either two or three times. Taking into account reported use of HCT services elsewhere (Figure 1b), of those attending all 3 seros, 48.0% never used HCT, 26.8% used HCT once and 25.3% used HCT two or more times. In total, of all participants attending at least one sero-survey (n = 17,483) and considering HCT use at sero-surveys and/or elsewhere, just 11.2% had ever tested twice or more.

**Figure 2:**
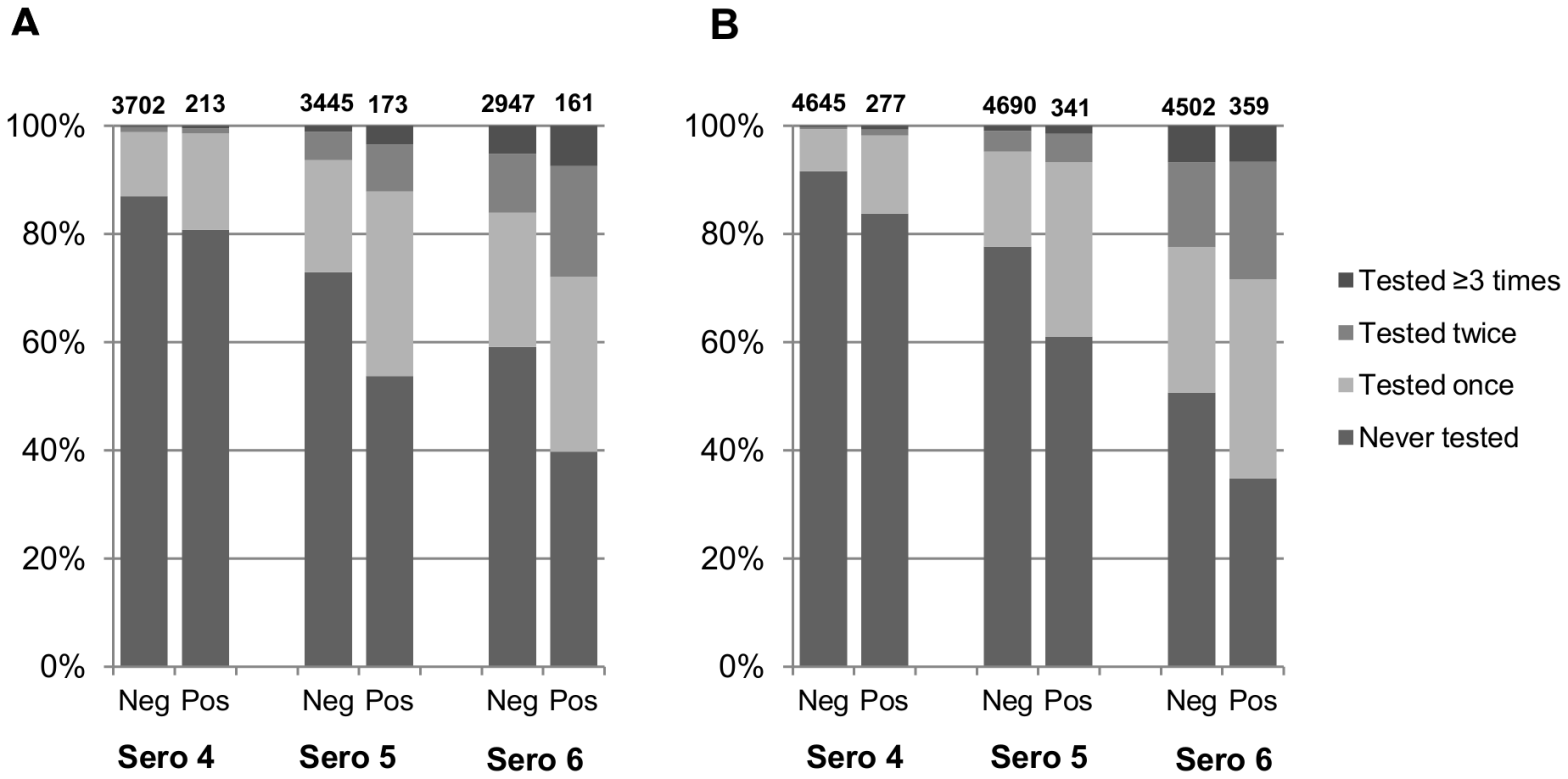
Number of times HCT was ever used (i.e. at sero-surveys and/or elsewhere) by the end of each sero-survey round by HIV status. Panel A) men; panel B) women. (NB: HIV status reflects status at the sero-survey round in question, rather than at any earlier test).

The proportion of participants who had never used any HCT service declined more rapidly over time among HIV-positive participants compared to HIV-negative. Figure 2 shows the proportions of men and women who had ever used HCT at sero-surveys and/or elsewhere by the end of each sero-survey round by HIV-status, with HIV-status reflecting status at the sero-round in question rather than at any earlier test. By 2010 (Sero-6), 60.2% of HIV-positive men and 65.2% of HIV-positive women had tested at least once, compared to 41.8% of HIV-negative men and 49.3% of HIV-negative women. Overall, 16.7% of men and 22.9% of women attending Sero-6 had ever repeat tested (i.e. tested twice or more).

When interpreting data which includes reported as well as known or documented use of HCT services, it should be noted that there is a tendency to both over and under report previous HCT service use. Table 4 shows that among HIV-negative individuals, of 7,015 instances where participants attended two consecutive sero-survey rounds (either Seros 4 and 5 and//or Seros 5 and 6), in 823 instances participants reported that they had used VCT at the earlier sero-survey round. However, comparison against earlier records revealed that in only 57.6% of cases (474/823) these participants *had* actually used VCT at the earlier round, with 42.4% (349/823) giving incorrect reports. Furthermore, of 822 instances where HIV-negative individuals had *known* use of VCT an earlier sero-survey round, in 42.3% of cases (348/822) these individuals failed to report this VCT use when asked at the later sero-survey round. Similar trends were seen among HIV-positive individuals, although in an even larger proportion of cases (49/89 or 55.1%) participants incorrectly reported VCT use at an earlier sero-survey round that did not actually occur. Of 67 instances where HIV-positive individuals had *known* use of VCT at an earlier sero-survey, in 40.3% of cases (27/67) participants failed to report this VCT use at the later round.

**Table 4:** By HIV status: number of times individuals reported using VCT at an earlier sero-survey round, versus number of times VCT was actually used at the earlier round.

HIV-negative at later sero-survey round	Reported at Sero-5 or 6 that used VCT at Sero-4 or 5, N (%)		
Actually used VCT at Sero-4 or 5	No	Yes	Total
No	5844 (94.4)	349 (42.4)	**6,193**
Yes	348 (5.6)	474 (57.6)	**822**
**Total**	**6192 (100)**	**823 (100)**	**7,015**
**HIV-positive at later sero-survey round**			
Actually used VCT at Sero-4 or 5	No	Yes	**Total**
No	328 (92.4)	49 (55.1)	**377**
Yes	27 (7.6)	40 (44.9)	**67**
**Total**	**355 (100)**	**89 (100)**	**444**

**Figure 3:**
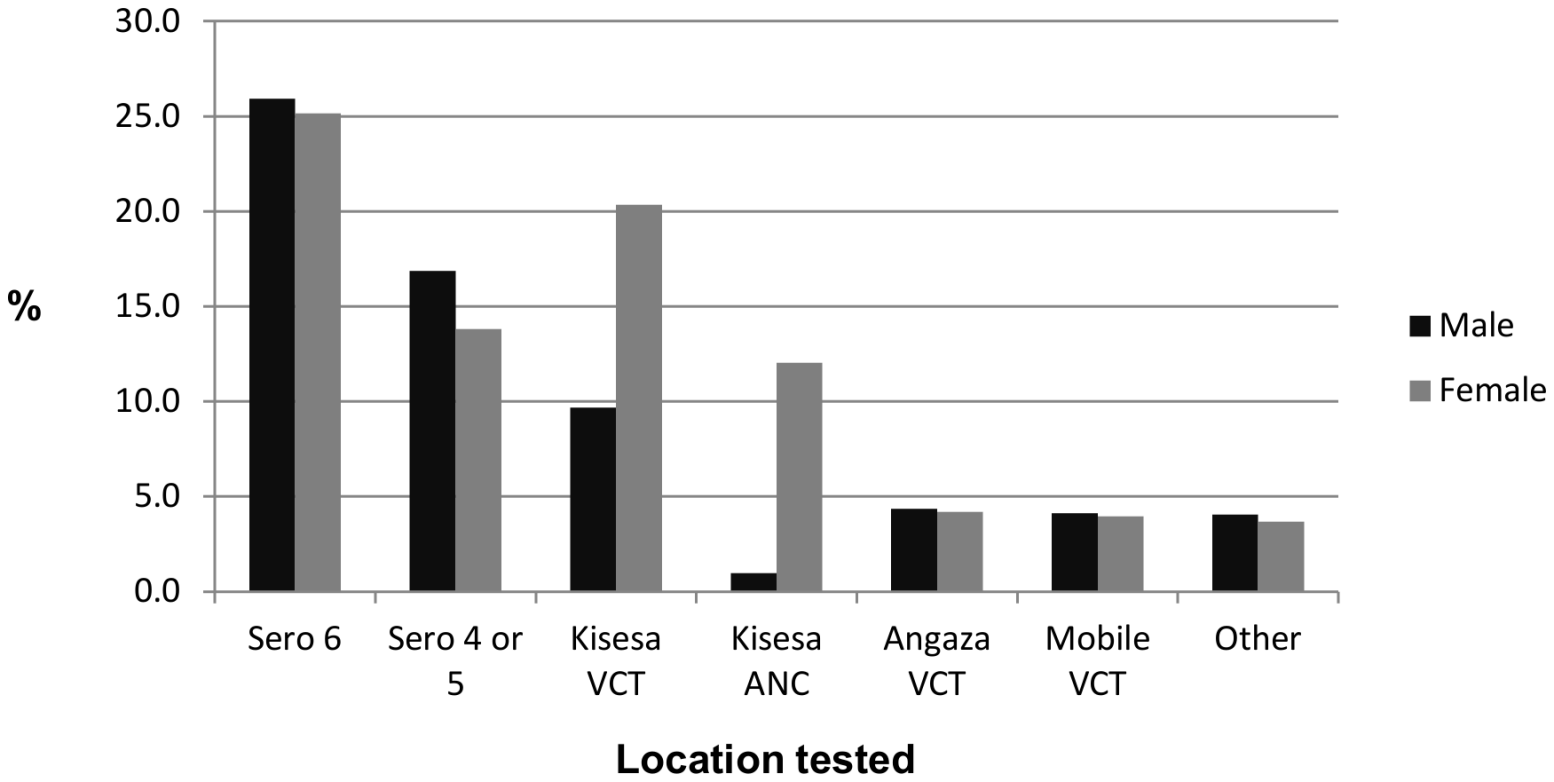
Among those who attended Sero 6, proportions of men (n = 3,131) and women (n = 4,877) using different HCT services.

Figure 3 shows the proportion of men and women attending Sero-6 who had used different HCT services. Outside of sero-surveys, the largest numbers of tests were carried out at the VCT and ANC clinics at Kisesa health centre. These clinics were used by a larger proportion of women than men. However, it is expected that only a small number of men would be tested at the ANC while attending with their partners. Smaller numbers of individuals tested at Angaza clinics in Mwanza city or elsewhere, at mobile VCT clinics and at other locations, where the proportionate use by men and women was more equal.

### Factors Associated with Repeat Use of VCT

Table 5 shows the characteristics of individuals who attended Sero-5 and used VCT at this round, and who also attended Sero-6 (n = 564; 234 men, 330 women). In total 321/564 participants (56.9%) used VCT at Sero-5 only, while 243/564 (43.1%) used VCT again at Sero-6 (i.e. repeat tested). Significantly fewer individuals who were HIV-positive at Sero-5 used VCT again at Sero-6 (n = 5/28, 17.9%) compared to those who were HIV-negative at Sero-5 (n = 238/534, 44.6%). However, a smaller proportion of individuals who sero-converted between sero-surveys (i.e. HIV-negative at Sero-5, HIV-positive at Sero-6) used VCT again at Sero-6 (n = 2/7, 28.6%) compared to those who did not sero-convert (n = 240/553, 43.4%). The proportions of individuals repeat testing increased with higher risk sexual behaviours (those married polygamously, those with higher numbers of sexual partners in their lifetime or in the last 12 months, and those using condoms inconsistently), although not all of these associations were statistically significant in univariable and multivariable analyses (Table 5).

**Table 5:** Characteristics of individuals attending both Sero-5 and Sero-6 and factors associated with repeat use of VCT at each round^#.^

		N	% Repeat Testing	OR (95% CI)	aOR (95% CI)
**Total**		**564**	**43.1**		
Sex	Male	234	45.7	1.2 (0.86**–**1.69)	**1.6 (1.03–2.47)** *
	Female	330	41.2	1	1
Age	15**–**24	99	42.4	1.25 (0.76**–**2.08)	
	25**–**34	150	45.3	1.41 (0.90**–**2.21)	
	35**–**44	142	48.6	**1.61 (1.03–2.53)** *	
	≥45	173	37.0	1	
Area of residence	Rural	294	32.7	1	1
	Roadside	176	53.4	**2.36 (1.61–3.47)** *	**2.67 (1.73–4.12)** *
	Trading Centre	94	56.4	**2.67 (1.66–4.29)** *	**2.34 (1.35–4.04)** *
Education	None	114	29.8	**0.48 (0.30–0.75)** *	**0.58 (0.34–0.98)** *
	Primary 1**–**4 yrs	44	36.4	0.64 (0.33**–**1.23)*	0.59 (0.27**–**1.25)
	Primary 5**–**7 yrs	312	47.1	1	1
	Secondary or higher	93	49.5	1.1 (0.69**–**1.75)	1.24 (0.67**–**2.27)
Religion	Catholic	256	41.4	1	1
	Other Christian	253	43.5	1.09 (0.77**–**1.55)	1.35 (0.91**–**2.01)
	Traditional	39	33.3	0.71 (0.35**–**1.44)	0.71 (0.31**–**1.65)
	Muslim	16	87.5	**9.91 (2.21–44.50)** *	**9.14 (1.88–44.47)** *
Marital status	Never married	66	45.5	1.14 (0.67**–**1.93)	0.72 (0.30**–**1.70)
	Married monogamous	348	42.2	1	1
	Married polygamous	67	52.2	1.5 (0.89**–**2.53)	**1.73 (0.97–3.08)** ¶
	Widowed	24	20.8	**0.36 (0.13–0.99)** *	0.45 (0.13**–**1.57)
	Separated/Divorced	50	44.0	1.07 (0.59**–**1.95)	1.45 (0.64**–**3.27)
Marital status change	No	373	42.1	1	
	Yes	91	39.6	0.9 (0.56**–**1.44)	
	Don’t know	100	50.0	1.38 (0.88**–**2.14)	
HIV status Sero-5	Negative	534	44.6	1	1
	Positive	28	17.9	**0.27 (0.10–0.72)** *	**0.17 (0.06–0.52)** *
Seroconverted Sero-5-Sero6	No	553	43.4	1	
	Yes	7	28.6	0.52 (0.10**–**2.71)	
BMI loss	None	363	44.6	1	
	<5%	106	40.6	0.85 (0.55**–**1.31)	
	≥5%	94	40.4	0.84 (0.53**–**1.34)	
Sero-4 VCT use	Didn’t attend Sero4	219	44.3	1.22 (0.85**–**1.75)	
	Attended Sero4 no VCT	263	39.5	1	
	Attended Sero4 used VCT	82	51.2	**1.61 (0.97–2.64)** ¶	
Spouse HIV status & VCT use^Δ^	No spouse identified	391	45.0	1	1
	Spouse HIV neg, no VCT	117	30.8	**0.54 (0.35–0.84)** *	**0.53 (0.31–0.91)** *
	Spouse HIV pos, no VCT	5	40.0	0.81 (0.13**–**4.93)	5.87 (0.68**–**50.52)
	Spouse HIV neg, used VCT	49	59.2	**1.77 (0.97–3.24)** ¶	1.27 (0.64**–**2.55)
	Spouse HIV pos, used VCT	1	0.0	**–**	**–**
Age at first sex	≥15	492	45.7	1	1
	<15	40	25.0	**0.4 (0.19–0.83)** *	**0.44 (0.19–1.02)** ¶
	Never had sex	13	23.1	0.36 (0.10**–**1.31)	**–**
	Don’t know	18	27.8	0.46 (0.16**–**1.30)	0.57 (0.17**–**1.88)
Number of sex partners in last year	None or one	450	41.6	1	
	Two	69	49.3	1.37 (0.82**–**2.27)	
	Three or more	33	54.5	1.69 (0.83**–**3.43)	
Lifetime number of sex partners	None or one	157	36.9	0.7 (0.45**–**1.08)	
	Two	99	43.4	0.92 (0.56**–**1.50)	
	Three or more	182	45.6	1	
	Don’t know	122	47.5	1.08 (0.68**–**1.71)	
Frequency of condom use^Ω^	Never use	361	42.4	1	1
	Inconsistent	98	57.1	**1.81 (1.15–2.85)** *	**1.78 (1.06–3.00)** *
	Consistent	14	50.0	1.36 (0.47**–**3.96)	1.04 (0.29**–**3.68)
	Never had sex	13	23.1	0.41 (0.11**–**1.51)	0.39 (0.08**–**1.77)
	Don’t know	78	30.8	0.6 (0.36**–**1.02)	0.55 (0.25**–**1.20)
Has a relative who is HIV-positive	No	355	40.6	1	
	Yes	171	51.5	**1.55 (1.08–2.24)** *	
	Don’t know	31	35.5	0.81 (0.37**–**1.73)	
Knows somebody taking ART	No	248	37.5	**0.64 (0.45–0.90)** *	
	Yes	276	48.6	1	
	Don’t know	32	50.0	1.06 (0.51**–**2.20)	

#Characteristics and behavioural variables are those reported at Sero-6, unless otherwise stated.

* p≤0.05,

¶ p≤0.1.

Δ Spouse HIV status and VCT use at Sero-6.

Ω Frequency of condom use in last 12 months considering three most recent partners.

The factors most strongly associated with repeat testing in multivariable analyses included area of residence (aOR for trading centre compared to rural villages: 2.34, 95% CI 1.35–4.04; aOR for roadside villages compared to rural villages: 2.67, 95% CI 1.73–4.12), being of Islamic faith (aOR for muslims compared to catholics: 9.14, 95% CI 1.88–44.47), testing HIV-negative at Sero-5 (aOR for testing HIV-positive compared to HIV-negative: 0.17, 95% CI 0.006–0.52), reporting using condoms inconsistently (aOR for using condoms inconsistently compared to never using condoms: 1.78, 95% CI 1.06–3.00) and being married polygamously (aOR for those married polygamously compared to those never married: 1.73, 95% CI 0.97–3.08). Those with no education were also significantly less likely to repeat test than those with a primary level of education (aOR 0.58, 95% CI0.34–0.98).

## Discussion

The uptake of HCT services increased steadily in Kisesa between 2003 and 2010, with 9.4% of all participants using VCT at Sero-4, 16.6% at Sero-5 and 25.5% at Sero-6. In spite of this, by the end of the Sero-6 round (n = 8,008), a substantial proportion of participants had never used any HCT service – approximately 36% of all HIV-positive individuals and 54% of all HIV-negative individuals. Furthermore, among participants who attended all three sero-survey rounds (n = 2,010), almost half (48.0%) had never used any HCT service (at sero-survey and/or elsewhere). This figure rose to almost two-thirds (64.0%) if considering VCT use during sero-survey rounds only.

Considering the rapid expansion of HIV testing and treatment services within the study area over the time period in question, it is disappointing that the proportions of individuals who had used an HCT service at least once were not even larger. Previous research has identified numerous barriers to the uptake of HIV testing services in sub-Saharan Africa including prohibitive costs associated with travelling to health clinics, shortages of test-kit supplies, high levels of stigma and discrimination and concerns about confidentiality [15]–[21]. It is likely that stigma formed the greatest barrier to VCT use during sero-surveys in this study, as transport was provided free of charge, test-kits were adequately supplied, and previous research has found trust in sero-survey counsellors to be high [14]. However, previous qualitative studies have found high levels of HIV-related stigma in Kisesa, which results in fear and a reluctance to test [22], [23]. Outside of sero-surveys, it is likely that a combination of the factors mentioned above contributed to the low rates of uptake of testing seen.

At each sero-survey round, those who had previously used an HCT service were significantly more likely to use the VCT service offered than those who had never used HCT before. However, among participants who attended all three sero-survey rounds (n = 2,010), the proportions repeat testing (i.e. using HCT twice or more) were low, with 11.1% using the VCT service offered at sero twice or more, and 25.3% having tested twice or more if reported use of HCT outside of sero-surveys was taken into account. As the latter estimate includes data on reported use of HCT services, we expect some error around it. However, the numbers of participants over and under reporting previous VCT use were very similar, with 399/917 (43.5%) of all individuals over-reporting previous VCT use that *did not* occur, and 378/896 (42.2%) under-reporting previous VCT use that *did* occur. As a result, the estimate is likely to provide a fairly accurate measure of the proportion of individuals who repeat tested among those who attended all three sero-survey rounds. While repeat HCT use did increase over time, the proportions testing more than once were still low by the end of the Sero-6 round, when 16.7% of all men and 22.9% of all women had ever repeat tested.

The proportions of participants repeat testing in Kisesa are somewhat lower than has been reported elsewhere in sub-Saharan Africa, where estimates ranged from 24.2% in a community cohort study in Uganda [11] to 73.4% in another cohort study in Malawi [10]. HCT services were provided in the home in both of these studies, which may have contributed to the higher rates of uptake seen. One recent systematic review found that home-based HCT may be one way in which to substantially increase the numbers of people undergoing HIV testing in sub-Saharan Africa [24], although such a service would likely place significant financial and logistical demands on the local health service infrastructure. In Kisesa, it is clear that HIV testing rates are currently well below the one test per adult per year required by Granich *et al* in their model [4], and further dramatic increases in the rate of uptake would be required if TasP were to be implemented.

In the analysis of factors associated with repeat VCT use (among those attending Seros 5 and 6), there was some evidence that HIV-negative people with higher risk sexual behaviours were among those who were most likely to repeat test. This is encouraging in terms of the potential to pick up those who are most at risk of HIV-infection. However, sero-converters were not significantly more likely to repeat test than those who did not sero-convert. Only 7 individuals sero-converted between the two rounds in this analysis, and so our sample size may have been too small to detect any meaningful difference. Other characteristics which were significantly associated with repeat VCT use included area of residence and level of education, highlighting a persistent inequity among those gaining access to HCT which was first seen in 2003–2004 [13].

Outside of sero-surveys, most participants reported having used HCT services at Kisesa health centre, where the VCT clinic was favoured disproportionately by women. This may reflect women who were offered PITC at the antenatal clinic but who were subsequently referred to the VCT clinic for testing (possibly due to shortages of staff or other resources at the antenatal clinic). However, it may also reflect a disinclination among men to use the health centre, which may be viewed as a place for women to receive services. Other HCT services such as mobile VCT clinics and the Angaza VCT clinic in Mwanza were used more equally by men and by women, but by smaller numbers of individuals overall, likely as a result of their temporary availability (mobile VCT clinics) or distance from the study area (Angaza clinic). With ever larger numbers of women being tested for HIV antenatally in sub-Saharan Africa [25], men may increasingly represent an under-served group in some settings.

This study has a number of strengths including a wealth of data covering an important period of time in terms of the development and increasing availability of HIV testing and treatment services in Kisesa. The study made use of data on both reported and actual use of HCT services, allowing for comparison of rates of testing using the two different data sources. It also allowed for comparison of the accuracy of reported versus actual HCT use, which was seen to be variable. This may have been caused by confusion when answering questions relating to previous HCT use and the exact location of any testing, and/or as a result of reporting biases.

We have seen declining participation rates in the Kisesa cohort study over time, particularly among younger men living in urban areas (Urassa *et al*, unpublished data), which may have affected our results. HIV-positive individuals may have been disproportionately represented among those not participating [26], which may have biased estimates of repeat testing upwards, as HIV-positive participants were seen to be less likely to repeat test in multivariable analyses. However, the main group of interest in terms of repeat testing is HIV-negative individuals. In contrast, estimates of repeat testing may have been biased downwards as a result of lesser participation among men living in urban areas, where people were significantly more likely to use HCT.

## Conclusions

Although the proportion of individuals using HCT services increased in Kisesa over time, further increases in the rates of first time and repeat HCT use would be required if TasP were to be implemented. Programmes and interventions designed to address the persistence of HIV-related stigma and discrimination are urgently needed, as are interventions designed to increase the uptake of HCT among those living in rural areas and those with least education.
